# Genetic testing of GCK-MODY identifies a novel pathogenic variant in a Chinese boy with early onset hyperglycemia

**DOI:** 10.1038/s41439-020-0096-0

**Published:** 2020-03-30

**Authors:** Kok-Siong Poon, Karen Mei-Ling Tan, Evelyn Siew-Chuan Koay, Andrew Sng

**Affiliations:** 1 Molecular Diagnosis Centre, Department of Laboratory Medicine, National University Hospital Singapore, Singapore, Singapore; 2 Department of Pathology, Yong Loo Lin School of Medicine, National University of Singapore, Singapore, Singapore; 3 Division of Paediatric Endocrinology, Department of Paediatrics, Khoo Teck Puat - National University Children’s Medical Institute, National University Hospital, Singapore, Singapore

**Keywords:** Genetic testing, Disease genetics

## Abstract

Glucokinase-maturity-onset diabetes of the young (GCK-MODY or MODY 2), caused by a heterozygous inactivating variant in the *Glucokinase* (*GCK)* gene, is a common form of MODY. Here, we present a case of GCK-MODY in a young Chinese boy, his sister and his father with a novel pathogenic variant in exon 8 of the *GCK* gene, NM_000162.5:c.1015del, p.(Glu339Argfs*14), which is predicted to cause a significant change in protein structure and function.

## Introduction

Maturity-onset diabetes of the young (MODY) or monogenic diabetes is an autosomal dominant form of non-insulin-dependent diabetes that typically presents before the age of 25 years^1^. GCK-MODY (OMIM #125851) is caused by a heterogeneous inactivating variant in the *Glucokinase* (*GCK*) gene^1^. The *GCK* gene is located on chromosome 7p15.3-p15.1 and consists of 10 coding exons that code for a 465-amino-acid protein, glucokinase, which is expressed in pancreatic beta cells, liver, and brain and regulated by tissue-specific promoters^2^. More than 600 different variants have been described and are distributed throughout the 10 exons and regulatory regions of the gene, without any “hot spots”^2^.

Glucokinase catalyzes the rate-limiting step of the glycolysis pathway, the conversion of glucose to glucose-6-phosphate, and functions as the glucose sensor in pancreatic beta cells^1,2^. Heterozygous inactivating variants in *GCK* reset the glucose threshold for insulin secretion to a higher level, resulting in fasting hyperglycemia^1^. Patients with GCK-MODY generally present asymptomatically with mild, stable fasting hyperglycemia and exhibit a normal return of glucose levels after an oral glucose load^1,2^. The identification of a *GCK* gene variant in a patient provides a definite diagnosis of GCK-MODY and helps to predict the likely prognosis and clinical course^2^.

**Fig. 1:**
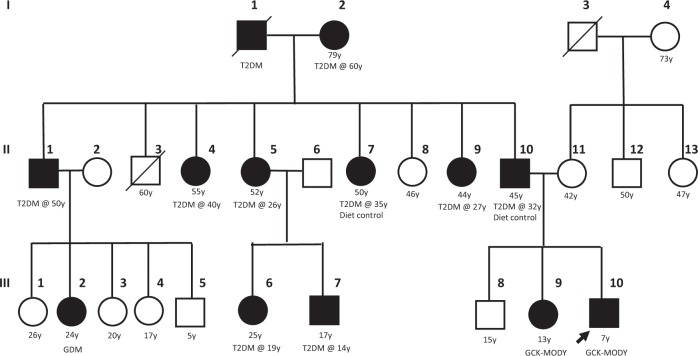
Family tree of the proband (III-10). Squares: male, circles: female. White: no T2DM, black: T2DM, GDM or GCK-MODY. GDM: gestational diabetes mellitus. The current ages of the family members are indicated below their symbols.

A 7-year-old boy (III-10) was referred to the pediatric endocrinology clinic for elevated random blood glucose of 9.8 mmol/L. The random blood glucose test was done at a routine health visit as he was overweight. He had no osmotic symptoms and was otherwise well. There is a strong family history of diabetes mellitus on the paternal side of the family (Fig. 1). His father (II-10) was diagnosed with type 2 diabetes mellitus (T2DM) when he was 32 years old and is on diet control. His first paternal aunt (II-4) was diagnosed with T2DM at 40 years old and is currently on oral medications. His second aunt (II-5) was diagnosed with T2DM at 26 years old and was started on oral medications as well, and both her children (III-6, III-7) were diagnosed with T2DM at 19 and 14 years old. His third aunt (II-7) was diagnosed with T2DM at 35 years old during her pregnancy. His fifth paternal aunt (II-9) was diagnosed with T2DM at 27 years old.

Upon examination, his height was 130.5 cm (90th percentile, +1.21 SDS), his weight was 38.95 kg (90th–97th percentile, +1.81 SDS), and his body mass index was 22.8 kg/m^2^ (95th–98th percentile, +1.75 SDS). He was prepubertal, and there were no clinical features of insulin resistance. A 2-h oral glucose tolerance test was performed, which showed a mild elevation in fasting blood glucose and a minor post-prandial excursion (6.5 to >8.7 mmol/L). His HbAlc was mildly elevated at 7.0% (>6.5%), and his fasting C peptide was normal at 423 pmol/L (364–1655 pmol/L).

Based on the strong family history of early-onset diabetes and the clinical and biochemical phenotype, monogenic diabetes was considered. GCK-MODY was the most likely differential; hence, no pharmacological treatment was started for this boy. Lifestyle counselling for healthy eating, exercise and weight loss was provided, and he remained well. He was reviewed in the outpatient clinic 6 months later, and his HbAlc remained stable at 7.3%.

Genetic testing for GCK-MODY was subsequently performed to confirm the clinical diagnosis in this boy. Genomic DNA was extracted from whole-blood samples using a LabTurbo DNA Mini kit (TAIGEN Bioscience Corporation, Taipei, Taiwan). The patient’s *GCK* gene (NM_000162.5) was screened for pathogenic variants. The promoter region, each of the 10 coding exons and flanking intron sequences of the gene were amplified by polymerase chain reaction (PCR) using the primers listed in Supplementary Table 1. PCR products were purified using a QIAquick gel extraction kit (Qiagen, Hilden, Germany) before being subjected to di-deoxy sequencing with a BigDye Terminator kit version 3.1 (Applied Biosystems, Austin, TX) on an ABI 3130 XL Genetic Analyzer (Applied Biosystems). Sequencing data were aligned to NCBI RefSeq NG_008847.2 using ATF software (Conexio Genomics, Fremantle, Australia) and Mutation Surveyor (SoftGenetics, city, PA).

**Fig. 2:**
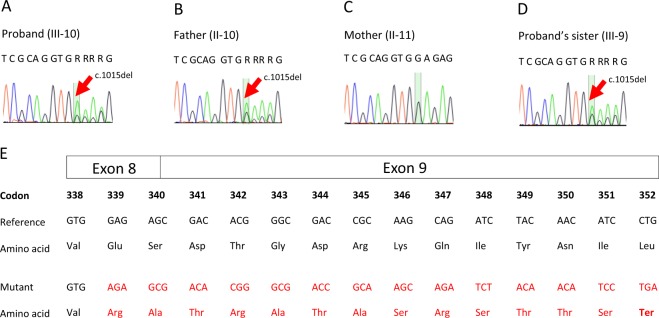
Partial nucleotide sequences of the GCK gene. Partial nucleotide sequences of the *GCK* gene of **a** proband, **b** proband’s father, **c** proband’s mother, and **d** proband’s sister. The red arrow indicates deletion of a G nucleotide of codon 339. **e** Protein translation from the normal (upper) and mutant (lower) nucleotide sequences are compared. A premature termination codon is predicted at codon 352.

A novel genetic variant NM_000162.5:c.1015del, p.(Glu339Argfs*14) of which the variant description information was verified with the Mutalyzer name checker^3^ was identified in exon 8 of the proband’s *GCK* gene (Fig. 2). This *GCK*:c.1015del, p.(Glu339Argfs*14) variant has not been previously reported in the literature, the public archive ClinVar, or curated databases, including the Human Gene Mutation Database (HGMD) and Leiden Open Variation Database (LOVD) 3.0. The variant was classified according to the American College of Medical Genetics and Genomics and the Association for Molecular Pathology (ACMG/AMP) standards and guidelines^4^. The deletion of a G nucleotide is predicted to cause frameshift in GCK protein translation and a change in protein length (PM4), resulting in loss of function of the gene product (PVS1) or a reduced level of functional glucokinase protein due to a nonsense-mediated decay mechanism. This variant was also not present in population databases such as the gnomAD database (>120,000 individuals), dbSNP and Exome Aggregation Consortium (PM2). In view of this evidence, this variant is classified as pathogenic in the context of GCK-MODY^5^.

The genetic testing results confirmed the diagnosis of GCK-MODY in this patient. His sister (III-9) presented subsequently to the pediatric endocrinology clinic at 12 years of age for impaired glucose tolerance. A 2-h oral glucose tolerance test was performed, which showed a mild elevation in fasting blood glucose and a minor post-prandial excursion (6.5 to >9.8 mmol/L). Her HbAlc was mildly elevated at 6.6% (>6.5%). She was overweight with a body mass index of 25.8 kg/m^2^. Cascade testing revealed that she and her father were heterozygous for the same *GCK*:c.1015del, p.(Glu339Argfs*14) variant as her brother. Both siblings were diagnosed at a young age and had fasting and postprandial glucose and HbA1c measurements consistent with GCK-MODY; thus, the variant is likely to cause autosomal dominant GCK-MODY inherited from the paternal family. Genetic testing for the variant is recommended for other affected family members to confirm the etiology of their diabetes. If GCK-MODY is confirmed, unnecessary pharmacological treatment may be eliminated. Fasting blood glucose measurement and presymptomatic/predictive genetic testing are also recommended for other at-risk family members. As MODY is an autosomal dominant condition, the patient’s offspring are at 50% risk of inheriting the variant and having fasting hyperglycemia. In this family, the proband’s sister will need to be managed during pregnancy in the future by serial monitoring of fetal growth by abdominal scans, with insulin therapy provided only if there is increased fetal growth^6^.

In summary, to our knowledge, this is the first report of GCK-MODY in a Chinese family caused by NM_000162.5(*GCK*):c.1015del, p.(Glu339Argfs*14), a novel pathogenic variant in exon 8. Genetic diagnosis helps to predict the clinical course and prognosis and guides the management of patients and their family members.

## Supplementary information

Supplemenatary table 1

## Data Availability

The relevant data from this Data Report are hosted at the Human Genome Variation Database at 10.6084/m9.figshare.hgv.2814.
