# Supplementary material for: Genetic testing of GCK-MODY identifies a novel pathogenic variant in a Chinese boy with early onset hyperglycemia
Source: Hum Genome Var. 2020 Mar 30;7:7. doi: 10.1038/s41439-020-0096-0 (PMC7105490; doi:10.1038/s41439-020-0096-0)
Supplement: Supplementary file 1 — Supplemenatary table 1 [file 41439_2020_96_MOESM1_ESM.docx]

**Supplementary Table 1** List of PCR and sequencing primers for *GCK* mutational analysis.

| Targeted *GCK* gene region | Forward | Reverse | PCR product size (base pairs) | Genomic coordinates (GRCh38.p12) |
| --- | --- | --- | --- | --- |
| Promoter, 5’UTR & exon 1 | atctgaacaggtggcaaagg | ccttctcaaagagcctgtgc | 867 | 44188798 .. 44189664 |
| Exon 2 | gtgtgcagatgcctggtg | ctggctgtgagtctgggagt | 346 | 44153207 .. 44153552 |
| Exon 3 | atatccgggctcagtcacct | ggccctgagatcctgcat | 300 | 44152216 .. 44152515 |
| Exon 4 | cggaagaggagagggaaact | agtttgagccccaccctact | 534 | 44150695 .. 44151228 |
| Exons 5 & 6 | tcagccctgcagaaatatga | ggctctgctctgacatcacc | 626 | 44149637 .. 44150262 |
| Exon 7 | ccattgttccagacaaagca | caagcccattatctgcaatg | 400 | 44147559 .. 44147958 |
| Exon 8 | gcacgttcctaatccctgac | cgccctgagaccaagtct | 437 | 44146387 .. 44146823 |
| Exons 9 & 10 | ggggcagtactaaccagtcc | caccgaaaaactgagggaag | 1000 | 44144956 .. 44145955 |
